# IMRT QA using machine learning: A multi‐institutional validation

**DOI:** 10.1002/acm2.12161

**Published:** 2017-08-17

**Authors:** Gilmer Valdes, Maria F. Chan, Seng Boh Lim, Ryan Scheuermann, Joseph O. Deasy, Timothy D. Solberg

**Affiliations:** ^1^ Department of Radiation Oncology University of California San Francisco Medical Center San Francisco CA USA; ^2^ Department of Medical Physics Memorial Sloan Kettering Cancer Center New York NY USA; ^3^ Department of Radiation Oncology Perelman School of Medicine University of Pennsylvania Philadelphia PA USA

**Keywords:** IMRT QA, machine learning, poisson regression, radiotherapy

## Abstract

**Purpose:**

To validate a machine learning approach to Virtual intensity‐modulated radiation therapy (IMRT) quality assurance (QA) for accurately predicting gamma passing rates using different measurement approaches at different institutions.

**Methods:**

A Virtual IMRT QA framework was previously developed using a machine learning algorithm based on 498 IMRT plans, in which QA measurements were performed using diode‐array detectors and a 3%local/3 mm with 10% threshold at Institution 1. An independent set of 139 IMRT measurements from a different institution, Institution 2, with QA data based on portal dosimetry using the same gamma index, was used to test the mathematical framework. Only pixels with ≥10% of the maximum calibrated units (CU) or dose were included in the comparison. Plans were characterized by 90 different complexity metrics. A weighted poison regression with Lasso regularization was trained to predict passing rates using the complexity metrics as input.

**Results:**

The methodology predicted passing rates within 3% accuracy for all composite plans measured using diode‐array detectors at Institution 1, and within 3.5% for 120 of 139 plans using portal dosimetry measurements performed on a per‐beam basis at Institution 2. The remaining measurements (19) had large areas of low CU, where portal dosimetry has a larger disagreement with the calculated dose and as such, the failure was expected. These beams need further modeling in the treatment planning system to correct the under‐response in low‐dose regions. Important features selected by Lasso to predict gamma passing rates were as follows: complete irradiated area outline (CIAO), jaw position, fraction of MLC leafs with gaps smaller than 20 or 5 mm, fraction of area receiving less than 50% of the total CU, fraction of the area receiving dose from penumbra, weighted average irregularity factor, and duty cycle.

**Conclusions:**

We have demonstrated that Virtual IMRT QA can predict passing rates using different measurement techniques and across multiple institutions. Prediction of QA passing rates can have profound implications on the current IMRT process.

## 1 INTRODUCTION

Over 50% of cancer patients receive radiotherapy as partial or full cancer treatment, and radiotherapy is an increasingly complex process. Machine learning is a subfield of data science that focuses on designing algorithms that can learn from and make predictions on data. Machine learning applications in radiotherapy have emerged increasingly in recent years, with applications including predictive modeling of treatment outcome in radiation oncology,^1^, ^2^, ^3^, ^4^, ^5^, ^6^, ^7^ treatment optimization,^8^, ^9^, ^10^, ^11^ error detection and prevention,^12^, ^13^, ^14^, ^15^ and treatment machine quality assurance (QA).^16^, ^17^, ^18^, ^19^ These machine learning techniques have provided physicians and physicists information for more effective and accurate treatment delivery as well as the ability to achieve personalized treatment.

To the best of our knowledge, however, little work with machine learning has been explored in the field of dosimetry and QA in clinical radiotherapy. It is common to perform patient‐specific pretreatment verification prior to intensity‐modulated radiation therapy (IMRT) delivery. This process is time consuming and not altogether instructive due to the myriad of sources that affect a passing result. In an earlier work, a machine learning algorithm, *Virtual IMRT QA*, was developed that can predict IMRT QA passing rates and identify underlying sources of errors not otherwise apparent.^20^ The algorithm identified the correlation between the IMRT plan complexity metrics and gamma passing rates and was validated on a single planning/delivery platform. The objective of this study is to further validate the approach using a large, heterogeneous dataset using different QA measurement devices (diode‐array detectors and portal dosimetry) on different models of treatment machines and at different institutions.

Identifying plans prone to QA failure allows physicists to concentrate resources in developing proactive approaches to QA and provides information on sources of errors needed to strategically improve the workflow of patient care as described in AAPM TG‐100.^21^ Goals of this study are to provide a framework to establish universal standards and thresholds, intercompare results, safely and efficiently implement adaptive radiotherapy, and in the long term, eliminate failing QA altogether. This represents a fundamental paradigm change in the way in which QA is performed.

## 2 MATERIALS AND METHODS

### 2.A Methodologies and data collection

**Figure 1:**
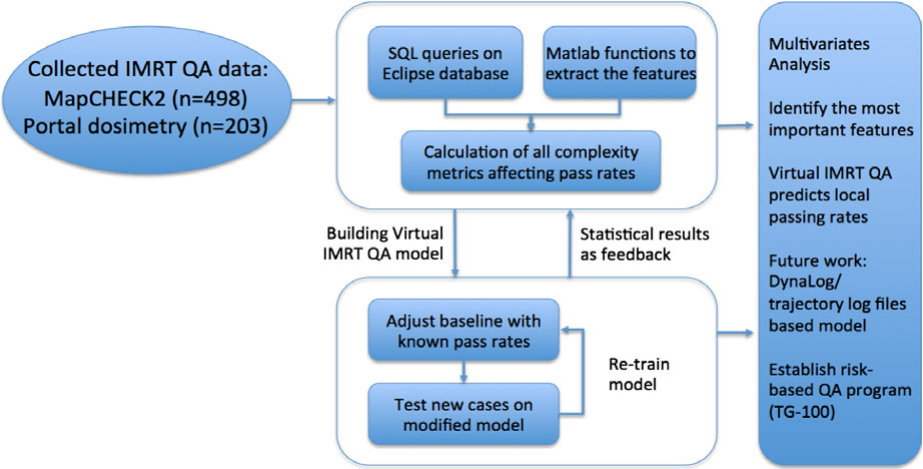
The workflow of the validation of *Virtual IMRT QA* model.

The *Virtual IMRT QA* framework was previously developed based on 498 IMRT plans, 416 and 82 using 6 and 15 MV, respectively, on TrueBeam and Clinac IX treatment units (Varian Medical Systems, Palo Alto, CA, USA), measured using a diode‐array detector (MapCHECK2, Sun Nuclear Corp) with a gamma criteria of 3%local/3 mm and 10% threshold. For each plan, parameters were extracted from the Eclipse treatment planning system (AAA, v11.0 – Varian Medical Systems, Palo Alto, CA, USA) using SQL queries. In order to further validate the framework, over 200 additional IMRT measurements (139 and 64 using 6 and 15 MV, respectively) based on Varian portal dosimetry (PD) on a Trilogy Linac at the Memorial Sloan Kettering Cancer Center (MSKCC) were tested using the same gamma criteria. The electronic portal imaging device (EPID) used in this study is a Varian aS1000 model with a pixel resolution of 0.392 mm and maximum image acquisition rate of 30 frames per second. The dark and flood field calibration along with a 10 × 10 cm, 100 MU at the source‐to‐detector distance of 105 cm were performed every day before QA to guarantee consistency of the measurements. Automatic registration was used to align the absolute dose distributions and analysis was performed using a gamma criteria 3%local/3 mm. For each IMRT beam, control point information was extracted from Eclipse v11.0 and different features that characterize the plans, Table 1, were calculated using Matlab Scripts, Matlab Inc, MA. Using all features and the corresponding passing rate, multivariate analysis was performed to identify the most important features that govern the passing rate. Poisson regression with Lasso regularization was trained using the new dataset acquired at MSKCC to learn the relation between the plan characteristics and each passing rate. Other details can be found in the original publication on the development of the Virtual IMRT QA method.^20^ Figure 1 summarizes the entire workflow of the construction and validation of the predictive IMRT QA model.

**Table 1:** Sample variables in Virtual IMRT QA modeling (continuous variables >90)

Number	Variable	Possible value
1	QA device	MapCHECK2, Portal dosimetry
2	Energy	6 MV, 15 MV
3	Machine type	TrueBeam, Trilogy, 21IX, 21EX, 6EX
4	Collimator angle	Mean value averaged over all control points
5	CIAO area (i.e., 5–>5 × 5)	<5, 5–10, 10–15, 15–20, 20–25, 25–30, >30
6	Jaw position	<5, 5–10, 10–15, 15–20, 20–25, >25
7	Small aperture score	Fraction of MLC gaps <2, 5, 10, 20 mm
8	Fraction of area receiving at least x% of CU	10, 20, 30, 40, 50
9	Irregularity factor	Fraction of area outside Radius = 5, 10, 20 cm
10	MLC leaf transmission	HD, M120‐pre‐2007, M120‐post‐2007
11	Perimeter	<10, 10–30, 30–50, 50–70, 70–90, 90–110, >110
12	Duty cycle (Total MU/Dose)	<2, 3–3, 3–4, 4–5, 5–6, >6
13	Modulation factor	Overall complexity (1, 2, 3)

For the portal dosimetry, only pixels with ≥10% of the maximum calibrated units (CU) or dose were included in the comparison. Plans were characterized by 90 different complexity metrics. A weighted poison regression with Lasso regularization was trained to predict passing rates using the complexity metrics as an input.

### 2.B Weighted Poisson regression with Lasso regularization

Let *S* = [(x_1_,y_1_), … (x_n_,y_n_)] be a set of paired data, where *y*_*i*_ is the number of detectors (pixels) that fail IMRT QA and *x*_*i*_ is a vector of complexity metrics of length 90 + 1 (as *x*_*1*_ is set to 1). Now let us assume that the number of measurement points that fail follows a Poisson distribution, as it is customary when counts are modeled:

(1) $$p\left(y=y_{i}|D_{i},fr\left(x_{i}\right)\right)=\frac{{\left(D_{i}*fr\left(x_{i}\right)\right)}^{y_{i}}e^{-D_{i}*fr\left(x_{i}\right)}}{y_{i}!}.$$

where *D*_*i*_ is the total number of detectors in the analysis and $fr\left(x_{i}\right)$ is the mean value of the failing rate of the plan i that depends on its complexity vector *x*_*i*_.

We can model *fr* according to a Poisson regression as:

(2) $$fr\left(x_{i}\right)=e^{\beta^{T}x_{i}}$$

where *β* is a constant vector the same size as *x*_*i*_

Now, given the realization of the data S, let us find the most likely vector *β*. In order to obtain *β*, we use Bayes theorem:

(3) $$p\left(\beta |S\right)=\frac{p\left(S|\beta \right)*p\left(\beta \right)}{p\left(S\right)}$$

where $p\left(\beta |S\right)$ is the posterior probability of *β* given S, $p\left(S|\beta \right)$ is the probability of obtaining S given *β*, $p\left(\beta \right)$ is the prior probability of *β*, and $p\left(S\right)$ is the probability of obtaining S regardless of *β*. We are interested in finding the *β* that maximizes the function $p\left(\beta |S\right)$, which is the same as:

(4) $$argmax_{\beta }p\left(\beta |S\right)=argmax_{\beta }\frac{p\left(S|\beta \right)*p\left(\beta \right)}{p\left(S\right)}=argmax_{\beta }\;p\left(S|\beta \right)*p\left(\beta \right)$$

In eq. 4 we have taken into account that $p\left(S\right)$ does not depend on β and as such, it can be dropped from the optimization problem. Assuming all measurements $\left(x_{i},y_{i}\right)$ are conditionally independent given the model, the probability $p\left(S|\beta \right)$ can be written as*:*

(5) $$p\left(S|\beta \right)=\prod_{i=1}^{n}\frac{{\left(D_{i}*e^{\beta^{T}x_{i}}\right)}^{y_{i}}e^{-D_{i}*e^{\beta^{T}x_{i}}}}{y_{i}!}$$

And assuming a Laplace distribution with a mean of 0 and variance equal to 2*λ* ^2^ for $p\left(\beta \right)$, as customary in Lasso regularization, we have

(6) $$p\left(\beta \right)=\frac{1}{2\lambda }e^{-\left|\beta \right|/\lambda }$$

which results in:

(7) $$argmax_{\beta }\;p\left(\beta |S\right)=argmax_{\beta }\left[\prod_{i=1}^{n}\frac{{\left(D_{i}*e^{\beta^{T}x_{i}}\right)}^{y_{i}}e^{-D_{i}*e^{\beta^{T}x_{i}}}}{y_{i}!}*\frac{1}{2\lambda }e^{-\left|\beta \right|/\lambda }\right]$$

As maximizing $p\left(\beta |D\right)$ is equivalent to maximizing log($p\left(\beta |D\right)$), eq. 7 can be rewritten as:

$$\begin{array}{cc} & argmax_{\beta }\log\left(p\left(\beta |S\right)\right)=\\ & argmax_{\beta }\log\left(\prod_{i=1}^{n}\frac{{\left(D_{i}*e^{\beta^{T}x_{i}}\right)}^{y_{i}}e^{-D_{i}*e^{\beta^{T}x_{i}}}}{y_{i}!}*\frac{1}{2\lambda }e^{-\left|\beta \right|/\lambda }\right) \end{array}$$

Applying the rules of logarithms and dropping the terms that do not depend on *β* results in:

(8) $$argmin_{\beta }\;\log\left(p\left(\beta |S\right)\right)=argmin_{\beta }\left[-\sum_{i=1}^{n}w_{i}\left(fr_{i}\beta^{T}x_{i}-e^{\beta^{T}x_{i}}\right)+\lambda \left|\beta \right|\right]$$

where *fr*_*i*_ is the observed failing rate for plan i, *w*_*i*_ = *D*_*i*_./*D*_*max*_ is a weight factor for each observation proportional to the number of detectors in the measurement and *D*_*max*_ is a normalization constant. Equation 8 is a weighted Poisson regression problem with Lasso regularization where *β* ^*T*^ can be obtained using the software package available at https://web.stanford.edu/~hastie/glmnet/glmnet_alpha.html.

Once *β* ^*T*^ is obtained, this constant vector is used together with eq. 2 and the complexity metrics of each plan *x*_*i*_ to predict a specific plan's passing rates as:

(9) $$fr_{i}=e^{\beta^{T}x_{i}}$$

## 3 RESULTS

**Figure 2:**
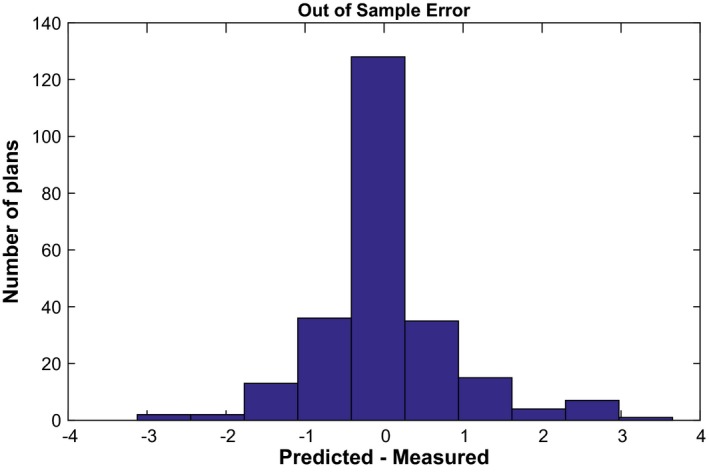
Residual error for Clinac and TrueBeam Linacs measured using MapCHECK2 at Institution 1.

**Figure 3:**
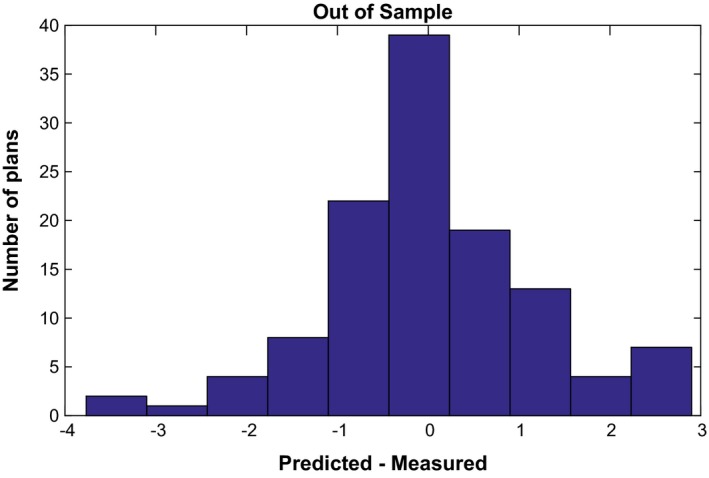
Residual error for a Trilogy (6 MV) at using portal dosimetry at Institution 2. Note that the inherent Varian's Portal Dose Image Prediction algorithm assumes a radially symmetric response which is certainly different than the reality in 2D profiles of portal dosimetry.^23^ This may add the additional uncertainty of this measurement.

Equation 9 was used to construct histograms of measured versus predicted passing rates using a 3%/3 mm local gamma threshold (Figs. 2 and 3). This magnitude commonly called residual is the standard metric to evaluate the performance of regression algorithm in a similar way that Area Under the Curve is used to evaluate performance in classification algorithms.^22^ All composite plans measured using diode‐array detectors were predicted within 3% accuracy^2^, while passing rates for portal dosimetry on per‐beam basis were predicted within <3.5% for 120 IMRT measurements delivered with 6 MV. The remaining measurements that used 6 MV (19) had large areas of low CU, where portal dosimetry exhibits poorer agreement with the calculated dose. This is due to the difference in response of an a‐Si electronic portal imaging device to open and MLC transmitted radiation, and further modeling using an algorithm such as that presented by Vial et al. can improve the under‐response in low‐dose regions.^23^, ^24^ These 19 IMRT beams with mean CU value of 0.127 ± 0.071 (range 0.022–0.254) have been excluded from the study pool. From the machine learning analysis, the important features selected by Lasso to predict gamma passing rates when the model was trained with MSKCC data were as follows: complete irradiated area outline (CIAO) area, jaw position, fraction of MLC leafs with gaps smaller than 20 or 5 mm, fraction of area receiving less than 50% of the total CU, fraction of the area receiving dose from penumbra, weighted average irregularity factor, and duty cycle among others. These features are the most likely feature to result in plans failing QA at different institutions that also use EPID dosimetry. Their specific quantitative contribution to the failing rate, however, will depend on the underlying model at each individual clinic. In addition, please note that all predictions are out of sample prediction, that is, the passing rates being predicted are not part of the data used to train the model.

**Figure 4:**
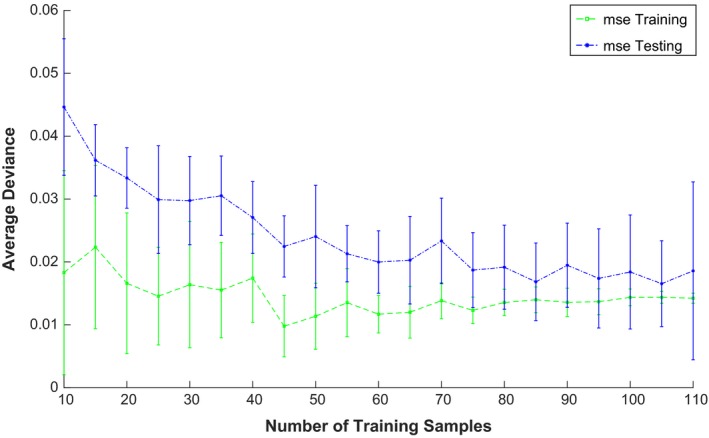
Learning Curve. Testing and Training error versus number of data points used to build the model.

Finally, a learning curve experiment was performed to estimate the number of plans needed in order for the model to converge to a solution. To evaluate confident intervals for the model, we constructed models by varying the number of training samples and calculating the training and testing error. The process was repeated 10 times and confident intervals calculated. Figure 4 shows that after approximately 80 data patients, no further improvement is obtained on average in the testing error. This number is roughly half the number obtained by Valdes et al.^20^ confirming that if the data are more homogeneous, that is, for Institution 2, only data from one Linac were analyzed, a smaller number of data points are needed to obtain a stable model.

## 4 DISCUSSION

An algorithm using machine learning, *Virtual IMRT QA*, has been developed to correlate the characteristics of IMRT plan and delivery characteristics and the corresponding gamma passing rate. With some adjustments on the features in the original model, Poisson regression with Lasso regularization was trained to learn the relation between the plan characteristics and each passing rate for different measurement devices. The predictive model was validated for different QA devices and methodologies on seven Linacs across two institutions. With our validation results, *Virtual IMRT QA* can predict gamma passing rates accurately within 3.5% as maximum empirical error observed. In addition, our previous work showed that 200 patient‐specific QA plans and results are required to train the virtual QA model to achieve an accurate prediction.^20^ The required training QA data should be readily available assuming a measurement‐based clinical patient‐specific QA program is in place, and should not impose additional measurement burden on the practicing physicist. The potential benefit of this approach can be quite significant. For instance, Virtual IMRT QA could be run by the dosimetrist while planning. If an arbitrary threshold of 93.5% for Virtual IMRT QA is set, all plans that satisfy this threshold should pass IMRT QA with a passing rate higher than 90%. These plans could be further measured. However, those that have predicted passing rate smaller than 93.5% could be modified without the need to perform the QA potentially saving valuable time. In addition to single gamma results, this model provides insight into factors contributing to the resulting failure points by identifying relevant features. This allows physicists to quantitatively assess different risk factors‐associated treatment plans. As TG‐100 calls for a new risk‐based QA program, this virtual QA model can be an invaluable tool to assist clinical physicists in their implementation. The virtual QA approach is not intended to replace measurement‐based QA, but rather to complement the measurement‐based program to provide a more comprehensive view. Through workflow improvement and risk analysis, the tool should enhance the overall QA and improve the safety of treatment delivery.

At present, the model is only capable of assessing fixed‐beam IMRT planning/delivery; features critical to volumetric‐modulated radiotherapy (VMAT) will be incorporated into future machine learning models. This should be acknowledged as a limitation of the current method. Analysis of 3D detectors will require the collection of some of the key delivery information, such as gantry speed, MLC speed, and aperture size, and obtaining these parameters posts potential challenges. With the popularity of this treatment modality, further study in this direction will be a great asset to the community. In addition, the predictive model was trained to correlate the automatic registration of calculated and measured QA doses which has its pros (uncertainty due to phantom misalignment is removed) and cons (some mechanical errors producing a shift of dose are not detected in QA).

In this study, we have used the formalism as described by Valdes et al.^20^ From eq. 9, however, it is clear that contributions from the different metrics were assumed to be linear (multiplication of a constant vector by the vector describing the plan characteristics), that is, no interaction terms between the different characteristics were considered. In addition, at the current stage, different models are needed for different combinations of delivery systems and energies. Ideally, one would like to incorporate all the data from an institution (delivery device, energy, QA devices, etc.) and input this data into a single mathematical formulation that will predict the gamma passing rate without the need for independent models which increases the data requirement. Even though beam data models for different Linacs/energies are different, they do share important characteristics and from an analytics point of view, it is inefficient to segregate the data by imposing practical constraints. We are currently developing a formulation that will take into account these limitations in order to obtain errors of local gamma passing rates within 2%. This number should be the ultimate goal of any quantitative analysis predicting gamma passing rate because as it has been reported by Nelms et al., some clinical relevant errors could create changes in gamma passing rate in the order of 2%.^25^ One possible way to improve accuracy within the current framework would be to separate data by treatment site and build a model for each treatment site. As in the case of different models for different energies, this approach will increase the need for more data. Finally, although we have decided to model here gamma passing rate with 3%/3 mm local analysis (for small targets 3%/2 mm might be more appropriate in the conventional QA), our methodology should not depend on the % dose or distance to agreement selected. That being said, at least in our dataset, stricter metric than 3%/3 mm results in having too much inherent noise for proper modeling.

## 5 CONCLUSIONS

In this work with more extensive QA data, the validity of *Virtual IMRT* QA to accurately predict gamma passing rates, within 3.5% error, has been shown for different models of Linacs in different institutions, providing a strong validation of our IMRT QA predictive model. Compared to conventional measurement‐based QA, the framework also provides significant insight into both machine and plan characteristics. Software‐based, *Virtual IMRT QA* using machine learning has a unique position in the radiotherapy QA program and further provides a framework for a future integrated risk‐based QA program such as that envisioned in AAPM TG‐100.

## CONFLICT OF INTEREST

The authors declare no conflict of interest.
